# Decoding glutamate receptor activation by the Ca^2+^ sensor protein hippocalcin in rat hippocampal neurons

**DOI:** 10.1111/j.1460-9568.2010.07303.x

**Published:** 2010-08

**Authors:** A V Dovgan, V P Cherkas, A R Stepanyuk, D J Fitzgerald, L P Haynes, A V Tepikin, R D Burgoyne, P V Belan

**Affiliations:** 1 Department of General Physiology of the Nervous System, Bogomoletz Institute of PhysiologyKiev, Ukraine; 2 Physiological Laboratory, School of Biomedical Sciences, University of LiverpoolLiverpool L69 3BX, UK

**Keywords:** NCS proteins, NMDA receptors, signal transduction, synapse, translocation

## Abstract

Hippocalcin is a Ca^2+^-binding protein that belongs to a family of neuronal Ca^2+^sensors and is a key mediator of many cellular functions including synaptic plasticity and learning. However, the molecular mechanisms involved in hippocalcin signalling remain illusive. Here we studied whether glutamate receptor activation induced by locally applied or synaptically released glutamate can be decoded by hippocalcin translocation. Local AMPA receptor activation resulted in fast hippocalcin-YFP translocation to specific sites within a dendritic tree mainly due to AMPA receptor-dependent depolarization and following Ca^2+^influx via voltage-operated calcium channels. Short local NMDA receptor activation induced fast hippocalcin-YFP translocation in a dendritic shaft at the application site due to direct Ca^2+^influx via NMDA receptor channels. Intrinsic network bursting produced hippocalcin-YFP translocation to a set of dendritic spines when they were subjected to several successive synaptic vesicle releases during a given burst whereas no translocation to spines was observed in response to a single synaptic vesicle release and to back-propagating action potentials. The translocation to spines required Ca^2+^influx via synaptic NMDA receptors in which Mg^2+^ block is relieved by postsynaptic depolarization. This synaptic translocation was restricted to spine heads and even closely (within 1–2 μm) located spines on the same dendritic branch signalled independently. Thus, we conclude that hippocalcin may differentially decode various spatiotemporal patterns of glutamate receptor activation into site- and time-specific translocation to its targets. Hippocalcin also possesses an ability to produce local signalling at the single synaptic level providing a molecular mechanism for homosynaptic plasticity.

## Introduction

Complex spatiotemporal changes in free cytosolic calcium concentration ([Ca^2+^]_i_) are decoded into changed activity of effector molecules by various Ca^2+^sensor proteins. A new emerging concept of cellular signal transduction suggests a dynamic model in which sensor proteins can translocate and undergo reversible binding interaction with the effector proteins rather than being solely activated by the rapid diffusion of Ca^2+^(Teruel & Meyer, 2000). Local signalling events have been well studied for Ca^2+^but studies of the molecular and cellular mechanisms underlying the signalling of Ca^2+^sensor proteins in living neurons have only began to emerge.

The neuronal calcium sensor (NCS) proteins, to which hippocalcin studied herein belongs, constitute a subfamily of EF-hand calcium-binding proteins mainly expressed in neurons and neuroendocrine cells (Burgoyne *et al.*, 2004). Recent studies have established their involvement in a wide range of Ca^2+^-dependent signalling processes including modulation and trafficking of ion channels, neurotransmitter release, synaptic plasticity, control of apoptosis and gene expression (McFerran *et al.*, 1998; Jinno *et al.*, 2004; Korhonen *et al.*, 2005; O'Callaghan *et al.*, 2005; Palmer *et al.*, 2005; Zheng *et al.*, 2005; Jo *et al.*, 2008). Information is also available regarding the involvement of NCS proteins in cancer, schizophrenia and several neurodegenerative disorders including Alzheimer's disease (Braunewell, 2005).

Hippocalcin possesses a Ca^2+^-myristoyl switch, a Ca^2+^-dependent conformation transition leading to protrusion of its myristoyl-containing hydrophobic N-terminal region out of a hydrophobic pocket (Ames *et al.*, 1997). This allows hippocalcin to translocate to membranes upon Ca^2+^binding that can be used in signal transduction processes (Kobayashi *et al.*, 1993; O'Callaghan *et al.*, 2003). Hippocalcin is highly expressed in hippocampal neurons, in particular in their dendrites, suggesting that it might be involved in postsynaptic signalling. It has been shown that Ca^2+^-dependent hippocalcin activation in hippocampal neurons is one of the necessary steps involved in expression of NMDA receptor (NMDAR)-dependent long-term depression (LTD) (Palmer *et al.*, 2005) and in production of a slow afterhyperpolarization (sAHP) (Tzingounis *et al.*, 2007). These and other hippocalcin-dependent signalling processes involve activation of postsynaptic glutamate receptors. However, it is still unknown if hippocalcin translocates in living hippocampal neurons in response to glutamate receptor stimulation. We hypothesized that complex changes in Ca^2+^concentration induced by glutamate receptor activation could result in hippocalcin signalling via its partitioning between a cytosol and specific sites at the plasma membrane in dendrites and spines of hippocampal neurons. In order to test this hypothesis we examined yellow fluorescent protein-tagged hippocalcin (hippocalcin-YFP) translocation induced in transiently transfected hippocampal cultured neurons by iontophoretically applied and synaptically released glutamate.

## Materials and methods

### Tissue cultures

All procedures used in this study were approved by the Animal Care Committee of Bogomoletz Institute of Physiology and conform to the Guidelines of the National Institutes of Health on the Care and Use of Animals. Neurons were obtained from newborn Wistar rats (age postnatal day 0–1; 56 animals of both sexes for the whole work) killed via rapid decapitation without anaesthesia. All rats were from the vivarium of Bogomoletz Institute of Physiology. Hippocampi of the rats were enzymatically dissociated with trypsin. The cell suspension (initial density of 3–5 × 10^5^ cells/cm^3^) was plated on glass coverslips coated with laminin and poly-l-ornithine (Invitrogen, Carlsbad, CA, USA). Cells were maintained in feeding solution consisting of minimal essential medium, 10% horse serum and N2 supplement (Invitrogen) in a humidified atmosphere containing 5% CO_2_ at 37°C as previously described (Melnick *et al.*, 1999).

### Plasmids

Hippocalcin-YFP, hippocalcin-CFP and enhanced yellow and cyan fluorescent protein (YFP, CFP) plasmids were prepared as described previously (O'Callaghan *et al.*, 2002).

### Transient transfection

Hippocampal neurons were transfected after 13–17 days in culture using Lipofectamine 2000 transfection reagent essentially as described by the supplier (Invitrogen). All cultures were used for the experiments 2–3 days after transfection. Transfection success was 0.2–1.0%.

### Electrophysiological recordings

Neurons growing in the cultures were visualized using inverted microscopes (IX70 or IX71; Olympus, Tokyo, Japan). Whole-cell patch-clamp recordings in either current- or voltage-clamp mode were obtained using an EPC-10/2 amplifier controlled by PatchMaster software (HEKA, Freiburg, Germany).

The composition of the extracellular solution was as follows (mm): NaCl 150, KCl 2, CaCl_2_ 2, MgCl_2_ 1, HEPES 10, glucose 10, glycine 0.01, pH 7.3, osmolarity 320 mOsm. Some experiments were carried out in the presence of d-2-amino-5-phosphonopentanoic acid (APV, 40 μm) or 6-cyano-7-nitroquinoxaline-2,3-dione (CNQX, 10 μm). If not indicated otherwise, gabazine (1 μm) or bicuculline (10 μm) were always present in the extracellular solution to block GABA_A_ receptors. The intracellular solution contained (mm): K-gluconate 118, KCl 30, NaCl 5, CaCl_2_ 0.3, EGTA 1, MgATP 2, GTP 0.3, HEPES 10, pH 7.3, osmolarity 290 mOsm. In some experiments conducted in voltage clamp mode, K^+^ was replaced with Cs^+^ and 3–5 mm QX-314, an intracellular sodium channel blocker, was added. Patch electrodes were pulled to obtain a resistance of 4–6 MΩ. Membrane voltage or transmembrane current were low-pass filtered (3 kHz) and acquired at 10 kHz. Recordings with a leak current > 200 pA or a series resistance of > 30 MΩ were discarded.

Iontophoretic glutamate application was performed via electrodes using a second channel of the EPC-10/2 amplifier. The iontophoretic electrodes were filled with a solution containing sodium glutamate (15–150 mm), HEPES (10 mm) and NaCl to give a final osmolarity of 310 mOsm, and to pH 7.3 with NaOH. Electrode resistance for the glutamate-containing solution was in the range 80–120 MΩ. Current pulses of 100 nA with a duration of 0.1–2.0 s were produced in current clamp mode in order to apply glutamate locally and a breaking current of 1–5 nA was used between the pulses to prevent leakage of glutamate. All experiments were conducted at room temperature.

### Hippocalcin-YFP translocation imaging

Time-lapse imaging of hippocampal neurons transiently transfected with fluorescent protein(s) was performed using a TILL Photonics wide-field imaging system (TILL Photonics, Gräfelfing, Germany) installed on inverted microscopes (IX70 or IX71, Olympus), using oil-immersion objectives (40 × , NA 1.35 or 60 × NA 1.25; Olympus), controlled by TILLvision software. An Imago CCD camera was used to precisely record fast changes in hippocalcin-YFP fluorescence, with acquisition rate typically in the range 0.5–2 Hz. A customized routine written in TILLvision software was used to calculate relative changes in hippocalcin-YFP fluorescence against initial baseline level in order to determine sites of hippocalcin-YFP fluorescence changes. Translocation, Δ*F*/*F*, was expressed as relative changes in hippocalcin-YFP fluorescence.

The following routine was used to determine translocation sites, over which regions of interest (ROIs) were placed and averaged values of hippocalcin-YFP fluorescence were calculated to demonstrate spatiotemporal patterns of hippocalcin-YFP translocation. Base and shifted movies were generated based on an initial movie recorded during imaging experiments. Each frame of the base movie was generated by averaging two to five frames of the initial movie, each frame of the shifted movie was generated by averaging the same number of frames (two to five) of the initial movie with a two- to seven-frame shift between base and shifted movies. The particular number of frames for averaging and shifting depended on the kinetics of hippocalcin-YFP translocation transients and acquisition frame rate. The base movie was subtracted from the shifted movie. The resulting differential movie was spatially filtered by an averaging filter with 3 × 3 kernels. Translocation sites were determined as simply connected regions with a level of hippocalcin-YFP fluorescence 3–4% higher than the baseline fluorescence. A green colour represents a decrease and red represents an increase in hippocalcin-YFP fluorescence.

Total hippocalcin-YFP fluorescence in a field of view was calculated for each frame during prolonged experiments and values of fluorescence in ROIs were normalized to the total fluorescence to compensate for protein photobleaching. In some experiments strong glutamate applications induced photobleaching-independent temporal decreases of hippocalcin-YFP fluorescence up to 5% of the initial value. This decrease was probably due to intracellular acidification (Micheva *et al.*, 2001) that resulted in a decrease of YFP fluorescence. When hippocalcin-CFP was used instead of hippocalcin-YFP, this decrease in fluorescence was almost negligible, confirming the relative independence of CFP fluorescence on pH compared with YFP. Because hippocalcin-YFP was mainly used as the hippocalcin sensor in this study and an acidification-dependent decrease of YFP fluorescence is not related to the protein translocation, we also normalized ROI values to the total hippocalcin-YFP fluorescence in a field of view to compensate for this decrease.

In order to observe hippocalcin-YFP translocation without substantial disturbance of intrinsic [Ca^2+^]_i_ regulation, we mainly studied translocation during the first 10-15 min after establishing a patch clamp configuration or in distal parts of a dendritic tree (100–350 μm from soma).

### Statistics

Quantitative results are presented as mean ± SEM, and statistical significance between groups was tested using directional (for antagonist applications) or non-directional (for comparisons of control and washout) Student's *t-*tests, with equal variances and a confidence level of 0.05. The mean for each experiment was calculated as the average for all neurons tested with a given protocol with the exception of experiments with spines. In the latter case, the mean was calculated as the average for all ROIs placed over spines.

### Chemicals

Glutamate and GABA_A_ receptor antagonists were obtained from Tocris (Bristol, UK) or Ascent Scientific (Bristol, UK). All other chemicals were purchased from Sigma (St Louis, MO, USA) and Invitrogen.

## Results

### Ionotropic glutamate receptor activation induces hippocalcin-YFP translocation

**Fig. 1:**
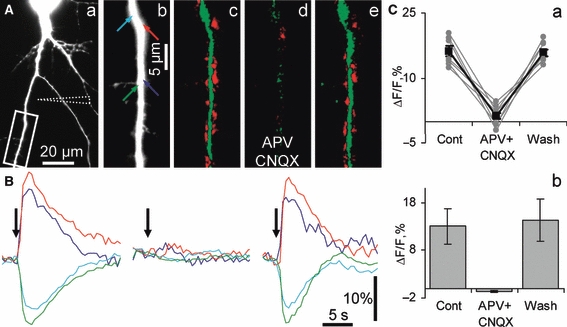
Local iontophoretic glutamate application induced hippocalcin-YFP translocation in cultured hippocampal neurons. (A) A set of images demonstrating glutamate-induced changes in hippocalcin-YFP fluorescence in an apical dendrite of an iontophoretically stimulated neuron. The fluorescent image (a) was taken using the YFP filter set. The position of the iontophoretic pipette is indicated by dashed lines. (Ab) A higher magnification image of a dendritic branch shown in the boxed area in Aa. Differential pseudocolour images were taken 2.5 s after the onset of short iontophoretic glutamate stimulation in control (c), in APV and CNQX (d), and after blocker washout (e). In this and other figures a green colour represents a decrease and red represents an increase in hippocalcin-YFP fluorescence. Colour arrows in b indicate sites where regions of interest (ROIs) were placed. Time courses of fluorescence changes in these ROIs in control, APV and CNQX, and after blocker washout are shown in B. Colours of traces match arrow colours in Ab. Onsets of iontophoretic glutamate applications are shown by black arrows. (C) Representative (taken from seven ‘red’ ROIs in the experiment shown in A) (a) and pooled (b) graphs showing a complete suppression of hippocalcin-YFP translocation by ionotropic glutamate receptor blockers.

Cultured rat hippocampal neurons were transfected to express hippocalcin-YFP. During time-lapse imaging, intact (not clamped) neurons expressing hippocalcin-YFP were stimulated locally with short (100–1000 ms, 100 nA) iontophoretic glutamate applications delivered from a glass microelectrode placed near an apical dendrite. The glutamate applications resulted in a fast (rise time of 1–2 s) hippocalcin-YFP translocation to a set of sites (sizes of 0.5–2.5 μm) on the dendritic tree (Fig. 1A and B). These sites were often located at dendritic branching points or initial parts of dendritic protrusions. At the same time, no translocation to heads of dendritic spines was observed. The translocation sites did not remain confined to the stimulation area but instead synchronously covered the whole dendritic tree in the field of view. The initial hippocalcin-YFP distribution was restored within 5–15 s after local stimulation and reproducible translocation to the same set of sites on the dendritic tree could be sequentially observed (Fig. 1A and B; *n* = 6 neurons). An increase in hippocalcin-YFP concentration in translocation sites was accompanied by a decrease in neighbouring sites, keeping the total value of hippocalcin-YFP fluorescence constant during a time course of translocation (Markova *et al.*, 2008). In neurons co-transfected to express hippocalcin-YFP and CFP or hippocalcin-CFP and YFP no changes in free CFP or YFP concentration in translocation sites were observed during the glutamate applications and respective hippocalcin-FP translocation (data not shown; see Markova *et al.*, 2008). The hippocalcin-YFP translocation was completely blocked by a cocktail of ionotropic glutamate receptor antagonists, CNQX (10 μm) and APV (40 μm) [16.3 ± 1.9% for control, −0.3 ± 0.4% under the blockers and 17.5 ± 2.6% in washout; control vs. blockers *t*= 8.6, *P*< 0.001; and vs. washout *t*= 0.38, *P*= 0.72; 46 ROIs, six cells; Fig. 1C). Thus, we conclude that the observed changes of hippocalcin-YFP fluorescence are ionotropic glutamate-receptor-dependent translocation of hippocalcin-YFP.

**Fig. 2:**
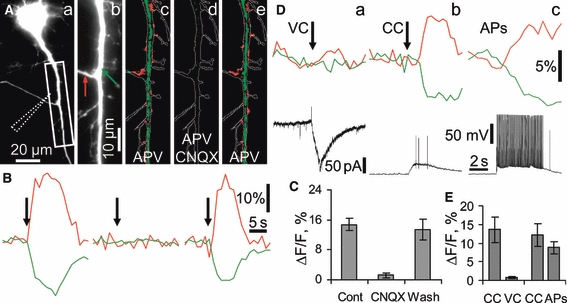
AMPARs activation resulted in hippocalcin-YFP translocation due to Ca^2+^influx via voltage-gated calcium channels. Experiments were conducted in the constant presence of APV (40 μm) in order to block NMDARs. (A) A set of images demonstrating AMPAR-dependent hippocalcin-YFP translocation in a neuron stimulated by iontophoretically applied glutamate. A fluorescent image (a) was taken using the YFP filter set. The position of the iontophoretic pipette is indicated by dashed lines. (Ab) A higher magnification image of a dendritic branch shown in a boxed area in Aa. Differential pseudocolour images were taken at 3 s after onset of iontophoretic glutamate application (1.0 s, 100 nA) in control (c) and CNQX-containing (d) solutions, and after CNQX washout (e). A green colour represents a decrease and red represents an increase in hippocalcin-YFP fluorescence. An outline of the dendritic tree is shown in each pseudocolour image for better visualization of translocation sites. Colour arrows in Ab indicate sites where ROIs were placed. Time courses of fluorescence changes in these ROIs in control, CNQX (10 μm), and after the blocker washout are shown in B. Colours of traces match arrow colours in Ab. Onsets of iontophoretic glutamate applications are shown by black arrows. (C) Pooled results demonstrating complete suppression of hippocalcin-YFP translocation by CNQX. (D) Hippocalcin-YFP translocation induced by different stimulation protocols: voltage (a) [−70 mV, (VC)] and current (b) clamp (CC) combined with iontophoretic glutamate application (1.0 s, 100 nA); intracellular stimulation with 100 bpAPs at 20 Hz (APs) with no glutamate application conducted (c). A red trace represents an ROI with an increase of hippocalcin-YFP fluorescence whereas a green one represents an ROI with a fluorescence decrease; black traces represent changes in membrane currents (a) and potential (b, c), respectively. (E) Pooled results showing a suppression of hippocalcin-YFP translocations in VC mode and comparable translocations in CC and bpAPs.

To check whether both AMPA receptors (AMPARs) and NMDARs contribute to hippocalcin translocation, we separately activated these types of receptors. The next series of experiments were conducted with APV (40 μm) to completely block NMDARs. A local iontophoretic glutamate application to an intact (not clamped) neuron induced fast, synchronous and reversible hippocalcin-YFP translocation to many sites in a soma and dendritic tree of a stimulated neuron that occurred within the whole field of view. As in the previous series of experiments, hippocalcin-YFP translocation to dendritic spines was not observed (Fig. 2A–C). The translocation was completely blocked by the AMPAR antagonist CNQX (10 μm) (15 ± 2% in control versus 1.2 ± 0.5% in presence of the antagonist; *t*= 11.6, *P*< 0.001; 65 ROIs, three neurons; Fig. 2C). Thus, AMPAR activation alone can induce hippocalcin translocation. The synchronous nature of this translocation in the very remote parts of the dendritic tree suggests that action potential (AP)-induced voltage-operated Ca^2+^channel (VOCC) activation may underlie the translocation. In line with this suggestion, AMPAR-dependent hippocalcin-YFP translocation was mainly abolished when the neuronal membrane potential was clamped at −70 mV (0.7 ± 0.3%; 38 ROIs, five neurons; Fig. 2D and E). The subsequent glutamate application to the same neurons held in current clamp mode induced substantial (to −40 to −20 mV) and prolonged (1–3 s) neuronal depolarization and burst of APs riding on the top of depolarization transients and a robust translocation (13.6 ± 3.4% compared with 0.7 ± 0.3% at −70 mV; *t*= 4.5, *P*= 0.001; 38 ROIs, five neurons; Fig. 2D and E). Trains of back-propagating APs (bpAPs) at 50 Hz with duration comparable with those of glutamate-induced depolarization resulted in hippocalcin-YFP translocation to the same sites. Pooled results showed that AMPAR-dependent translocation in current clamp mode was insignificantly larger than bpAP-induced translocation (12.2 ± 3.1% during AMPAR activation versus 8.8 ± 1.7% during bpAP trains; *t*= 0.9, *P*= 0.18, non-directional test; 64 ROIs, ten neurons; Fig. 2D and E).

In order to validate the role of VOCCs in AMPAR-dependent hippocalcin-YFP translocation, we carried out experiments in which AMPA was iontophoretically applied to neurons held in current clamp mode (*V*_m_∼−60 to 70 mV) in the presence of APV (40 μm). As in the experiment shown in Fig. 2Db, the applications induced neuronal depolarization and a burst of APs riding on the top of the depolarization and hippocalcin-YFP translocation. When Cd^2+^(200 μm) was washed into the extracellular solution the AMPA-induced translocation was almost completely abolished (10.1 ± 1.1% in control versus 1.3 ± 1.4% in the presence of Cd^2+^; *t*= 4.3, *P*= 0.0026; 21 ROIs, four neurons) whereas neuronal depolarization was preserved.

Thus, the experiments described above clearly show that most of the AMPAR-dependent hippocalcin-YFP translocation is due to the indirect action of glutamate resulting in AMPAR-dependent depolarization and VOCC activation.

**Fig. 3:**
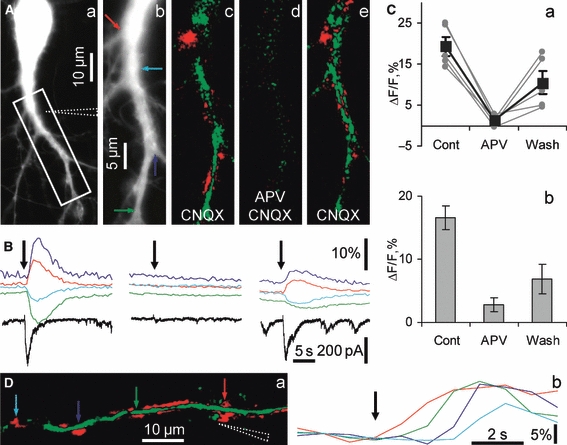
Hippocalcin-YFP translocated due to direct Ca^2+^influx via NMDARs. (A) Images demonstrating NMDAR-dependent hippocalcin-YFP translocation in a neuron stimulated by iontophoretically applied glutamate in Mg^2+^-free solution in the presence of CNQX (10 μm), gabazine (5 μm) and glycine (10 μm). A fluorescent image (a) was taken using the YFP filter set. The position of the iontophoretic pipette is indicated by dashed lines. (Ab) A higher magnification image of a dendritic branch shown in the boxed area in Aa. Differential pseudocolour images were taken at 2.5 s after an onset of iontophoretic glutamate application (0.5 s, 100 nA) in control (c) and APV-containing (d) solutions, and after APV washout (e). A green colour represents a decrease and red represents an increase in hippocalcin-YFP fluorescence. Colour arrows in b indicate sites where ROIs were placed. Time courses of fluorescence changes in these ROIs in control, APV (40 μm) and after blocker washout are shown in B. Colours of traces match arrow colours in Ab. Onsets of iontophoretic glutamate applications are shown by black arrows in B. The currents (black traces) were recorded in voltage clamp mode at -60 mV to abolish Ca^2+^influx via VOCC and leave NMDARs as the only source of Ca^2+^influx. (C) Representative (taken from five ‘red’ ROIs in the experiment shown in A) (a) and pooled (b) graphs showing strong suppression of hippocalcin-YFP translocation by APV. (D) Hippocalcin-YFP translocation due to local activation of NMDARs and site-specific association of hippocalcin-YFP with the plasma membrane. A diffusional wave of glutamate released from a pipette (shown by dashed lines in a) during an iontophoretic pulse (200 ms, 100 nA; onset of application is indicated by a black arrow in b) initially induced hippocalcin-YFP translocation in a dendritic branch in a site proximal to the pipette (red arrow), after that in a more distal site indicated by a green arrow and finally in the most distal sites (blue and cyan arrows). Colour coding of traces in (b) matches the colours of arrows in (a). The distance from the pipette tip to the most distal ROI is about 50 μm and the glutamate wave passed this distance for about 3 s, in agreement with an estimated rate of glutamate diffusion in the extracellular solution. There was no translocation in more distal dendritic sites, indicating that glutamate did not reach NMDARs in these sites.

In the next step glutamate was iontophoretically applied to neurons in the presence of an AMPAR antagonist, CNQX (10 μm). Recordings were performed in Mg^2+^-free extracellular solution in order to relieve NMDARs from Mg^2+^block. In neurons clamped at −60 mV, glutamate application induced an inward current and spatially restricted hippocalcin-YFP translocation to many sites. Both the translocation and current were completely blocked by the NMDAR antagonist APV (40 μm) [16.6 ± 2.8% in control versus 2.8 ± 1.1% with the antagonist (*t*= 6.4, *P*< 0.001); 222 ± 37 pA in control vs. 27 ± 7 pA with the antagonist (*t*= 5.2, *P*< 0.001) for the translocation and current, respectively; 53 ROIs, five neurons; Fig. 3A–C). Hippocalcin initially translocated to sites proximal to the iontophoretic electrode and translocation events then spread along the dendrite at 20–30 μm/s (Fig. 3D; five neurons; at least two applications to each neuron), which is close to the rate of glutamate diffusion in the extracellular solution. Thus, direct activation of synaptic and extrasynaptic NMDARs by a diffusion wave of glutamate and respective Ca^2+^influx to the dendritic shaft is probably the only reason for the observed hippocalcin-YFP translocation to certain sites of the dendritic plasma membrane.

As shown above, no translocation in response to glutamate application was observed in a cocktail of AMPAR and NMDAR blockers. This suggests that metabotropic glutamate receptor activation alone, which may result in inositol 1,4,5-triphosphate (IP_3_)-dependent Ca^2+^mobilization in hippocampal neurons, did not induce [Ca^2+^]_i_ elevations that are high enough to produce hippocalcin signalling via its translocation. Nevertheless, metabotropic receptors might contribute to intracellular Ca^2+^mobilization when Ca^2+^influx is induced by ionotropic glutamate receptor activation as IP_3_ receptors are both Ca^2+^- and IP_3_-dependent (Bezprozvanny, 2005). Therefore, glutamate-induced hippocalcin-YFP translocation was compared in extracellular solutions lacking or containing the metabotropic glutamate receptor antagonist α-methyl-4-carboxyphenylglycine (MCPG) (300 μm). No significant changes in spatio-temporal patterns and amplitudes of translocation were observed in neuronal dendritic trees under study (12.0 ± 4.4% without MCPG vs. 11.4 ± 4.4% during antagonist perfusion; *t*= 0.1, *P*= 0.46; 23 ROIs, four neurons; data not shown), indicating that metabotropic glutamate receptor activation does not contribute to hippocalcin signalling, at least in the case of local stimulation.

The other mechanism that may take part in hippocalcin translocation during glutamate stimulation is Ca^2+^-dependent Ca^2+^release from the endoplasmic reticulum (ER) (Bardo *et al.*, 2006). However, no changes in hippocalcin-YFP translocation in the dendritic tree was observed when cultures were incubated with the ER Ca^2+^-ATPase blocker cyclopiazonic acid (CPA, 10 μm) (8.3 ± 2.3% in control vs. 7.4 ± 1.2% during CPA perfusion and 8.5 ± 0.9% in washout; *t*= 0.34, *P*= 0.37; 26 ROIs, four neurons).

Thus, we have shown above that NMDARs are the only glutamate receptors whose local activation induces site-specific hippocalcin translocation. Taking into account that activity-dependent release of glutamate in glutamatergic synapses should lead to synaptic NMDAR activation we aimed to check if such activation might result in hippocalcin translocation.

### Synaptic NMDAR activation induces local spine-specific hippocalcin translocation

**Fig. 4:**
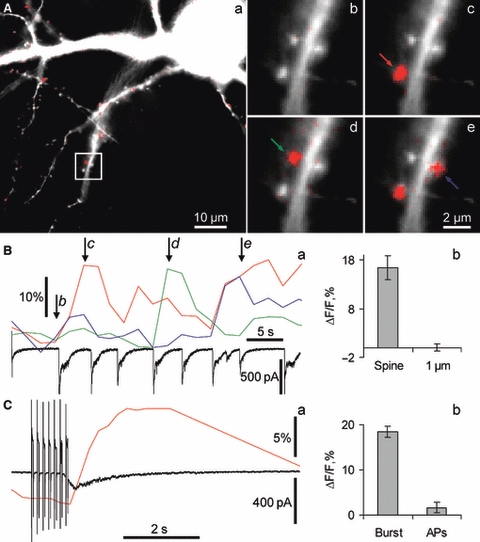
Strong activation of synaptic NMDARs induced hippocalcin-YFP translocation to dendritic spines. (A) An overlay of morphological (white) and hippocalcin-YFP translocation (red) images of neuron during a spontaneous burst of synaptic NMDAR-dependent currents at the time indicated as *d* in Ba. All synapses that were active during the burst appear in red. Panels b–e demonstrate overlays of morphological (white) and translocation images taken at the times indicated by respective letters in italic in Ba. Colour arrows indicate spines for which time courses of hippocalcin-YFP translocation are demonstrated in Ba. NMDAR-dependent currents were simultaneously recorded in whole-cell voltage clamp mode (holding potential −70 mV) and shown in Ba (black trace). (Bb) Values of hippocalcin-YFP translocation to spines compared with those in a dendritic tree at 1 μm from the respective spines. (C) Hippocalcin-YFP translocation to spines in response to membrane depolarization and to NMDAR-dependent synaptic current. An example of hippocalcin-YFP fluorescence changes and simultaneously recorded transmembrane current is shown in Ca. In this example, a spontaneous NMDAR-dependent postsynaptic current developed due to a network burst immediately after the train of depolarizing pulses (seven pulses from −70 to 0 mV; 50 ms at 7 Hz). It is clear that the current via synaptic NMDARs rather than the train induced hippocalcin-YFP translocation. In all neurons tested with this particular protocol no translocation was observed as a result of the trains. (Cb) Pooled results showing that a vigorous bpAP train (100 APs at 20 Hz) did not lead to hippocalcin-YFP translocation to sites where bursting-induced translocation was observed. At the same time, the trains did induce hippocalcin-YFP translocation to neighbouring sites in the dendritic shaft. ROIs were only placed over sites where bursting-induced hippocalcin-YFP translocation was observed. Experiments were conducted with CNQX, gabazine and glycine and without Mg^2+^.

Neurons in the hippocampal cultures under study revealed both miniature and activity-dependent excitatory postsynaptic potentials (EPSPs) with one or a few APs riding on the top of fast EPSPs. Neither of these two types of spontaneous synaptic activity resulted in measurable hippocalcin-YFP translocation, indicating that this neuronal Ca^2+^sensor is able to filter low-frequency synaptic and AP activity. Reducing [Mg^2+^] below physiological levels induces enhanced excitability in both hippocampal slices (Walther *et al.*, 1986) and cultures (Mangan & Kapur, 2004). In our hands in a nominally zero external Mg^2+^medium with a GABA_A_ receptor blocker, gabazine (1 μm), and an AMPAR blocker, CNQX (10 μm), neurons demonstrated recurrent spontaneous AP bursts superimposed on prolonged depolarization. In voltage clamp mode (−70 mV) this spontaneous network activity resulted in bursts of inward postsynaptic currents completely blocked by APV (40 μm) (data not shown), indicating that synaptic NMDARs are most probably involved in the generation of these postsynaptic currents. Since NMDARs are relieved of Mg^2+^block in Mg^2+^-free solution, they are probably the only source of Ca^2+^that could potentially induce hippocalcin translocation in pyramidal neurons clamped at −70 mV. During imaging of neuronal dendritic trees having numerous spines, we observed spontaneous hippocalcin-YFP translocation to a set of spines correlated with bursts of postsynaptic currents (Fig. 4, supplementary Movie S1; 18.9 ± 1.0%, 149 translocation events in 65 spines of eight neurons). Each burst induced hippocalcin-YFP translocation to slightly or completely different set of spines that were active during the respective burst. Hippocalcin-YFP translocation to dendritic shaft locations was also rarely observed. The translocation and bursts of postsynaptic current were completely blocked by APV (40 μm; data not shown). The translocation into spines was tightly spatially restricted to a spine head with no significant changes in hippocalcin-YFP fluorescence in a dendrite at the beginning of the spine neck (16.2 ± 2.1% in spines vs. 0.1 ± 0.7% near the neck, *t*= 6.5, *P*< 0.001; 35 translocation events in 19 spines, three neurons; Fig. 4B b) or adjacent dendritic membranes (data not shown). Spines that were separated by a few micrometres on the dendritic tree signalled independently (Fig. 4, Movie S1). Thus, strong activation of single synapses, which induces synaptic NMDAR-dependent Ca^2+^influx, is sufficient to mediate homosynaptic hippocalcin signalling in conditions when the Mg^2+^block of the channels is relieved.

### Other sources of [Ca ^2+^]_i_ mobilization do not result in hippocalcin translocation to spines

We also assessed whether [Ca^2+^]_i_ increase induced by VOCC activation alone may result in hippocalcin-YFP translocation to dendritic spines. It has been shown that the proximal dendrites of hippocampal pyramidal neurons are different from spines in the expression of different types of VOCC (Yasuda *et al.*, 2006) with substantial contribution to bpAPs of T-, L- and N-types of calcium channels whereas R-type dominate in the latter structures (Bloodgood & Sabatini, 2007). Activation of VOCC channels as a result of bpAPs or membrane depolarization lead to an increase in [Ca^2+^]_i_ in dendritic spines (Bloodgood & Sabatini, 2007) and in theory should result in hippocalcin translocation to these structures.

Two different protocols to selectively activate VOCC were used in this series of experiments. First, we applied series of depolarizing stimuli from −70 to 0 mV (10–50 ms at 7–20 Hz; five neurons) with a total duration of 1 s resembling the duration of the bursts. These stimuli did not produce translocation to spines whereas spontaneous bursts occurring in the same neurons did (Fig. 4Ca). We have previously shown that short bpAP trains (ten APs at 20 Hz) do not induce substantial hippocalcin-YFP translocation in a dendritic tree of hippocampal neurons (Markova *et al.*, 2008). Therefore, we suggested that the applied stimulation could be too mild to induce translocation to spines. Thus, in the next round of experiments neurons revealing spontaneous bursting were challenged with a train of bpAPs (100 APs at 20 Hz) and bpAP-induced hippocalcin-YFP translocation was quantified in spines, in which translocation was induced during the bursts. No translocation was observed in such sites as a consequence of bpAP trains (18.4 ± 1.2% during the bursts and 1.6 ± 1.1% during APs; *t*= 12.7, *P*< 0.001; 42 ROIs, five neurons; Fig. 4Cb). At the same time, these bpAP trains induced robust hippocalcin-YFP translocation to neighbouring sites in a dendritic shaft in these (data not shown) and other neurons (Fig. 2D and E).

**Fig. 5:**
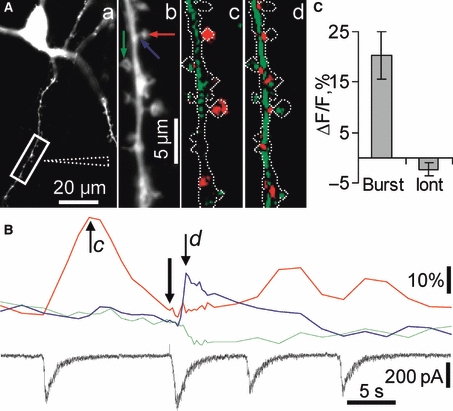
Activation of synaptic and total pools of NMDARs resulted in differential hippocalcin-YFP signalling in spines. Spatial patterns of hippocalcin-YFP translocation induced by activation of synaptic and total (synaptic and extrasynaptic) pools of NMDARs. The synaptic pool was activated during spontaneous network bursts whereas the total pool was stimulated by iontophoretic glutamate application to a neuronal dendritic branch. (A) A fluorescent image (a) was taken using the YFP filter set. The position of the iontophoretic pipette is indicated by dashed lines. (Ab) A higher magnification image of a dendritic branch shown in the boxed area in Aa. Ac and Ad demonstrate translocation images taken at the times indicated by respective letters in italic in B. These differential pseudocolour images were taken after an onset of network burst (c) and of iontophoretic glutamate application (0.5 s, 100 nA) (d). An outline of the dendritic tree is shown in each pseudocolour image for better visualization of translocation sites. A green colour represents a decrease and red represents an increase in hippocalcin-YFP fluorescence. Colour arrows in Ab indicate sites where ROIs were placed. Time courses of fluorescence changes in these ROIs are shown in B. Colours of traces match arrow colours in Ab. An onset of iontophoretic glutamate application is shown by a black arrow in B. The postsynaptic current (black trace) was recorded in voltage clamp mode at −70 mV to abolish Ca^2+^influx via VOCC and leave NMDARs as the only source of Ca^2+^influx. (C) Pooled results demonstrating that hippocalcin-YFP translocates to dendritic spines during synaptic rather than both synaptic and extrasynaptic NMDAR activation. Experiments were conducted with CNQX, gabazine and glycine and without Mg^2+^in an extracellular solution in order to isolate NMDAR-dependent currents and to relieve them from Mg^2+^block.

We also tested if simultaneous activation of synaptic and extrasynaptic NMDARs with a temporal profile analogous to that observed during bursting activity generated hippocalcin translocation to spines. To isolate NMDARs from VOCC, neurons were incubated in Mg^2+^-free media containing CNQX (10 μm) and voltage clamped at -70 mV. We imaged dendritic branches during spontaneous network bursts and during short iontophoretic glutamate applications to dendritic branches under study. Both types of stimulation induced analogous glutamate receptor-dependent postsynaptic currents (Fig. 5) that were completely blocked by APV (data not shown). At the same time, hippocalcin-YFP translocation patterns were substantially different for these types of stimuli. Network bursting resulted in hippocalcin-YFP translocation to spines (two spines in an example shown in Fig. 5Ac) whereas glutamate iontophoresis mainly induced translocation to many neighbouring sites in the dendritic shaft rather than to spines (Fig. 5Ad). Translocation in the dendritic shaft appears obvious given that extrasynaptic NMDAR activation should increase [Ca^2+^]_i_ in the dendritic shaft, thereby inducing hippocalcin-YFP translocation in this dendritic compartment. Nevertheless, it is interesting to note that hippocalcin-YFP translocation to spines was practically abolished in spite of synaptic NMDAR activation during glutamate application (20.2 ± 4.7% during the bursts versus −2.4 ± 1.3% during glutamate application, *t*= 4.6, *P*= 0.002; 33 spines, four neurons; Fig. 5), implying that only particular patterns of synaptic NMDAR activation may lead to hippocalcin signalling into spines.

Thus, the specific spatiotemporal pattern of [Ca^2+^]_i_ changes induced by the intrinsic pattern of synaptic NMDAR activation in the dendritic tree of hippocampal neurons is preferentially decoded by hippocalcin translocation to the dendritic spines and no other Ca^2+^sources can trigger this type of hippocalcin signalling.

### Hippocalcin translocation to spines during bursting neuronal activity

**Fig. 6:**
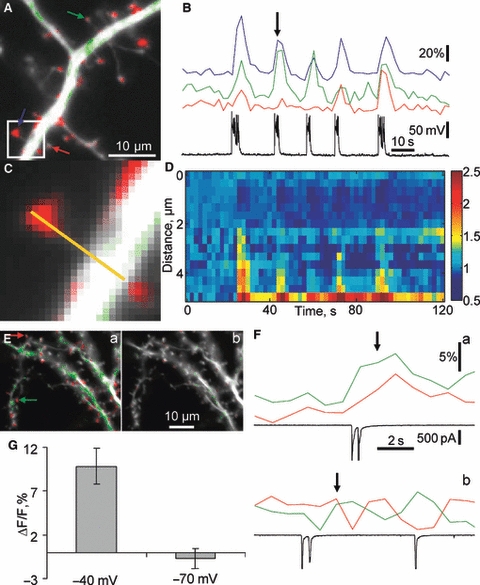
Intrinsic bursts of network activity induced hippocalcin-YFP translocation to dendritic spines. (A) An overlay of hippocalcin-YFP fluorescent image and pseudocolour image demonstrating hippocalcin-YFP translocation sites at the moment indicated by a black arrow in B. A green colour represents a decrease and red represents an increase in hippocalcin-YFP fluorescence. Colour arrows in A indicate spines in which changes of fluorescence during bursting activity are demonstrated in B with the same colour coding. Changes in the neuronal membrane potential were simultaneously recorded and shown in B as a black trace. The translocation occurred in a normal extracellular solution (no glutamate receptor blockers and 1 mm Mg^2+^) with 1 μm of gabazine to decrease inhibition in order to induce bursting network activity. Not all spines showed an increase in hippocalcin-YFP fluorescence in response to a particular spontaneous network burst (compare red and green traces in B). A part of the dendritic tree indicated as a white square in A is shown in C at higher magnification. (D) Time course of hippocalcin-YFP fluorescent changes along a yellow line in C (distance ‘0’ represents a yellow line end opposite to a dendritic head). (E, F) Hippocalcin-YFP translocation to spines was due to direct Ca^2+^influx via NMDARs rather than due to other mechanisms related to the bursting network activity. Hippocalcin-YFP translocation induced by spontaneous bursting activity was recorded in a neuron voltage clamped at −40 mV (Ea, Fa) and −70 mV (Eb, Fb) in order to relieve and engage Mg^2+^block of NMDARs, respectively. (E) An overlay of morphological (white) and hippocalcin-YFP translocation images of a neuron taken at the time indicated by black arrows in F. Colour arrows in Ea indicate spines in which fluorescent changes during bursting activity are demonstrated in F with the same colour coding. Changes in the neuronal membrane current were simultaneously recorded and shown in F as black traces. (G) Comparison of hippocalcin-YFP translocation amplitudes in spines at −40 and −70 mV.

As shown above, hippocalcin-YFP translocation to dendritic spines occurs when synaptic NMDARs are the only source of Ca^2+^to trigger this process, but this might not be the case during a normal pattern of activity when a neuron is depolarized and/or it generates APs simultaneously with NMDAR activation as takes place during the bursts. VOCC Ca^2+^influx occurring during the burst together with NMDAR-dependent Ca^2+^influx and the ER Ca^2+^stores produce another spatiotemporal profile of [Ca^2+^]_i_ that in its turn may result in hippocalcin translocation to other membrane targets. Therefore, we tested whether hippocalcin-YFP translocation to spines is physiologically relevant and can occur during native patterns of neuronal activity (Ben Ari, 2001). Neurons were incubated in an extracellular solution with a higher Mg^2+^concentration (1 mm) that is enough to engage Mg^2+^block of NMDARs (Monyer *et al.*, 1994), and GABA_A_ receptor blockers. Although we observed changes in bursting activity, the bursts could still be reliably induced in a proportion of cultures (most probably due to block of inhibitory synaptic transmission by GABA_A_ receptor blockers). Time-lapse imaging of transfected neurons showed that robust hippocalcin-YFP translocation to dendritic spines was still present during the bursts (Fig. 6A–D) but not during subthreshold and low-frequency spontaneous activity (data not shown). Translocation to the plasma membrane of dendritic shafts due to simultaneous NMDAR and VOCC activation during the bursts was more frequently observed in this case (red spots in Fig. 6A and C) compared with recordings in voltage clamp mode when it was relatively rare (Fig. 4A and B). Hippocalcin-YFP fluorescence was simultaneously decreased in certain areas of the dendritic tree (green areas in Fig. 6A and C), indicating sites from which hippocalcin-YFP was diffusionally translocated.

The level of translocation was not equal within a given spine head, with some pixels showing a several-fold increase in hippocalcin-YFP fluorescence (Fig. 6C and D), indicating that hippocalcin-YFP might be inserted in suboptical (in terms of size) areas of plasma membrane and reaching an extremely high level of concentration there. The amplitude of translocation transients to spines was comparable with that observed in voltage clamp mode in Mg^2+^-free extracellular solution (16.9 ± 0.8%, 153 translocation events, 21 spines, five neurons; compare Figs 4B and 6B). In line with previous results, translocation was blocked by APV (75 translocation events in 19 spines during 14 bursts in controls, no translocation in the same set of spines during 44 bursts in APV).

Although a block of hippocalcin-YFP translocation to spines by APV suggests involvement of NMDARs in the triggering of this type of synaptic signalling, it is possible that APV acted by generally changing the excitability of the neuronal network rather than specifically blocking a critical trigger for translocation. Therefore, we tested whether preventing postsynaptic NMDAR activation via Mg^2+^block by voltage clamping cells at −70 mV during the bursts would decrease the translocation. This manipulation prevented the translocation to spines (−0.7 ± 1.1%, four neurons; Fig. 6Eb, Fb and G). When the same cells were voltage clamped at −40 mV, spontaneous bursting resulted in hippocalcin-YFP translocation to spines (9.8 ± 2.1 vs. −0.7 ± 1.1% at −70 mV; *t*= 4.4, *P*= 0.002; 17 ROIs; four neurons; Fig. 6Ea, Fa and G).

As in the case of Mg^2+^-free extracellular solution (Fig. 4C), trains of bpAPs (100 stimuli at 20 Hz) led to hippocalcin-YFP translocation in the dendritic shaft rather than to the spines (supplementary Fig. S1). A significant difference was found when hippocalcin-YFP translocation in response to bpAPs was quantified in the morphologically clearly defined spines, revealing hippocalcin-YFP translocation in response to the bursts (13.3 ± 1.3% during the bursts and −0.6 ± 1.4% during bpAPs; *t*= 6.7 *P*= 0.0013; nine spines, four neurons; Fig. S1). Thus, activation of VOCCs does not result in hippocalcin translocation to the dendritic spines. These results demonstrate that hippocalcin may signal to different locations of the dendritic tree depending on patterns of neuronal activity, thus decoding these patterns into site-specific activation of its plasma membrane targets.

Different types of VOCC as well as non-linear interaction between R-type VOCC and NMDARs (Bloodgood & Sabatini, 2007) may contribute to [Ca^2+^]_i_ mobilization in dendritic spines initiated by synaptic NMDAR activation. We used L- and R/T-type channel antagonists [nimodipine (10 μm) and mibefradil (10 μm), respectively], in the extracellular solution to block VOCC that may substantially contribute to Ca^2+^influx to the spines during the bursts. We could not block P/Q- and N-type VOCCs as they secure glutamate release in presynaptic terminals; fortunately, they do not substantially contribute to [Ca^2+^]_i_ transients in the spines (Bloodgood & Sabatini, 2007). The VOCC antagonists did not induce depression of hippocalcin-YFP translocation to spines during the bursts (16.9 ± 0.8% in control and 18.3 ± 1% in the antagonists; *t*= 1.1, *P*= 0.26, non-directional test; 48 spines five neurons), implying that synaptic NMDARs are probably the main source of Ca^2+^influx to spines resulting in hippocalcin translocation.

## Discussion

Previous studies have demonstrated that hippocalcin signalling is necessary for spatial and associative memory (Kobayashi *et al.*, 2005) and plays an important role in synaptic plasticity (Palmer *et al.*, 2005) and many other cellular functions (Lindholm *et al.*, 2002; Tzingounis *et al.*, 2007). However, the cellular mechanisms involved in hippocalcin signalling remain illusive. This is in part because of the lack of temporal resolution of existing experimental approaches (mainly based on immunocytochemical assays) for studies of fast molecular movement and interaction in living cells. Here we used hippocalcin tagged with fluorescent proteins to study hippocalcin translocation induced by fast glutamate receptor activation. By combining electrophysiology with hippocalcin-FP imaging we showed that hippocalcin may differentially decode glutamate receptor activation and that network bursting discharges result in homosynaptic hippocalcin signalling in active dendritic spines.

### Dynamic range of hippocalcin concentration changes in the plasma membrane

A major consideration in determining the intrinsic dynamics of hippocalcin translocation is that overexpression of recombinant protein should not substantially change its initial pool size and stoichiometry of interaction with target proteins. The endogenous hippocalcin concentration in hippocampal pyramidal cells was estimated to be more than 30 μm (Furuta *et al.*, 1999). This concentration may be the highest among the EF-hand-type calcium-binding proteins in these cells, and is comparable with calmodulin concentration (Kakiuchi *et al.*, 1982). Although we did not take any precautions to limit the exogenous protein pool, expression levels comparable with 30 μm are normally not observed in mammalian cells and rarely exceed 10–20 μm (van der Wal *et al.*, 2001). Thus, the exogenous hippocalcin pool was hardly above 50% of the endogenous pool. Also, qualitatively similar results regarding hippocalcin translocation to spines were obtained in neurons with different (up to five-fold) levels of hippocalcin expression, indicating that overexpression was not crucial for its intrinsic dynamics.

Amplitudes of hippocalcin-YFP translocation transients ranged from several per cent up to 60% of the initial value of hippocalcin-YFP concentration (average value of about 20%). Thus, the dynamic range of changes in hippocalcin concentration in spines appears to be narrow. However, actual changes of hippocalcin concentration at its site of action – the plasma membrane – might be substantial, allowing hippocalcin to act as a fine tuning regulator of its membrane targets. In order to estimate the dynamic range of hippocalcin we need to determine its concentration in the plasma membrane at rest and during bursting. It has been suggested that hippocalcin is mainly a cytosolic protein (Kobayashi *et al.*, 1993; Markova *et al.*, 2008). This implies that a very small fraction of the hippocalcin molecules (if any) are present in the plasma membrane at the resting level of [Ca^2+^]_i_. To estimate the amount of hippocalcin-YFP associated with the spine plasma membrane during the burst we took into account the following considerations. First, the spatial distribution of hippocalcin-YFP in the dendritic shaft adjacent to active spines and, consequently, cytosolic hippocalcin-YFP concentration, was not changed when neurons were clamped at −70mV during the burst (Fig. 4Bb). Second, hippocalcin-YFP translocation to spines lasted substantially longer (∼5–10 s; Fig. 4Ba) than the characteristic time necessary for molecules of hippocalcin size to diffusionally equilibrate via a spine neck (< 1 s) (Majewska *et al.*, 2000; Sharma *et al.*, 2006). Based on the observations above we suggest that the cytosolic hippocalcin concentration in the spines and adjacent dendritic shaft is approximately equal at the peak of translocation transients and is the same as before the translocation. Thus, in the first approximation all the hippocalcin translocated to dendritic spines (up to 60% of resting level) should be associated with the plasma membrane where just a few per cent of the total hippocalcin could be present at rest. In other words, hippocalcin concentration in the plasma membrane of spines can be increased dozens of times during robust hippocalcin translocation. Taking into account that hippocalcin can translocate to diffractionally limited spots within the plasma membrane of spines rather than being randomly associated with the plasma membrane during the translocation (Fig. 6D) the dynamic range of hippocalcin concentration changes in the plasma membrane might be even wider, allowing hippocalcin to act as a fine-tuning regulator of intracellular signalling events.

### Biophysical mechanisms of hippocalcin translocation

**Fig. 7:**
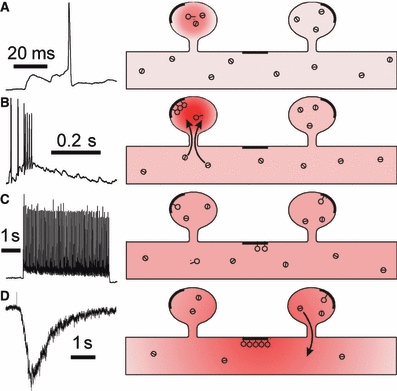
Hippocalcin translocation differentially decodes distinct patterns of [Ca^2+^]_i_ changes induced by glutamate receptor activation. Schematic of [Ca^2+^]_i_ changes and hippocalcin translocation in a part of the dendritic tree. The respective neuronal electrical activity is shown in the right part of the figure. Deeper hues of red show higher [Ca^2+^]_i_ levels. Circles with a dash inside and outside denote free and Ca^2+^-bound forms of hippocalcin, respectively. Thicker lines show high-affinity plasma membrane sites. (A) Single subthreshold and threshold EPSPs induce [Ca^2+^]_i_ transient limited to a specific spine with no visible hippocalcin translocation. (B) Several glutamate vesicles successively released in the same active synapse during the burst induce a larger and longer [Ca^2+^]_i_ transient leading to robust hippocalcin translocation only to such spines. (C) A train of bpAPs cannot form the spatiotemporal pattern of [Ca^2+^]_i_ necessary for hippocalcin translocation to the spines, rather resulting in lower and slower (compared with burst-induced spine translocation) hippocalcin accumulation in high-affinity (‘sticky’) plasma membrane sites of the dendritic tree. (D) Glutamate application induces local activation of synaptic and extrasynaptic NMDARs. The respective local Ca^2+^influx results in hippocalcin translocation to ‘sticky’ dendritic sites in this local dendritic area. A decreased cytosolic hippocalcin concentration in the dendritic tree prevents hippocalcin translocation to the spines in spite of synaptic NMDAR activation.

One of the major findings from this study is that there is no obvious direct relationship between Ca^2+^influx and hippocalcin translocation; for example, translocation does not occur in response to both short trains of APs (Markova *et al.*, 2008) and short activation of synaptic NMDARs in spite of the fact that Ca^2+^influx via VOCCs and NMDARs increases [Ca^2+^]_i_ (Sabatini *et al.*, 2002; our unpublished results). Translocation to spines is not observed during vigorous AP stimulation in spite of robust Ca^2+^influx via VOCCs. Even prolonged synaptic NMDAR activation during glutamate iontophoretic application does not result in hippocalcin translocation to spines. A complex interplay between [Ca^2+^]_i_ changes, Ca^2+^-dependent hippocalcin transitions between its different states, the distribution of local plasma membrane affinities for hippocalcin, the interaction of hippocalcin with target proteins and particular morphology of dendritic segments in determining hippocalcin and Ca^2+^diffusion results in a specific pattern of hippocalcin translocation. Thus, it is hard to see how hippocalcin may locally translocate in response to a certain pattern of neuronal activity and experimental measurements are possibly the only way to determine this. Nevertheless, some simple considerations might be taken into account to qualitatively describe the biophysical mechanisms underlying the observed translocation of hippocalcin. Here we show that Ca^2+^influx via synaptic NMDARs is the main mechanism inducing robust hippocalcin translocation to dendritic spines, whereas other sources of [Ca^2+^]_i_ increase, for example OCC activation by bpAPs, were ineffective in this role. A spatiotemporal profile of [Ca^2+^]_i_ in the spines should be considered in order to explain this apparent contradiction. Although each AP can generate a large [Ca^2+^]_i_ transient in the spines, a rapid clearance of Ca^2+^(about 12 ms; Sabatini *et al.*, 2002) should prevent a large [Ca^2+^]_i_ accumulation in the spines during AP trains used in this study (Fig. 7C). Also, an on-rate of Ca^2+^binding for many EF-hand-type Ca^2+^-binding proteins is reported to be low (Holmes, 2000) and therefore these proteins may be too slow to follow fast [Ca^2+^]_i_ transients induced by APs. As AP-evoked [Ca^2+^]_i_ transients in the spines remain at high levels for just several milliseconds they should be ineffective in activating the Ca^2+^-myristoyl switch of hippocalcin (Fig. 7C). Therefore, hippocalcin translocation to the spines was not observed in these conditions.

At the same time, a single vesicle of glutamate released in a particular synapse (Fig. 7A) resulted in a local [Ca^2+^]_i_ increase in spines without substantial [Ca^2+^]_i_ increase in the dendritic shaft (Sabatini *et al.*, 2002). A precise compartmentalization of [Ca^2+^]_i_ signals together with its relatively long duration and high amplitude (∼100 ms and ∼10 μm; Sabatini *et al.*, 2002) could result in hippocalcin translocation. Nevertheless, this local [Ca^2+^]_i_ transient did not induce translocation to spines, indicating that the [Ca^2+^]_i_ increase was either not high or not long enough for hippocalcin to bind Ca^2+^and to induce the Ca^2+^-myristoyl switch. It is possible that as in the case with calmodulin, the most studied EF-hand-type protein, higher [Ca^2+^] (above 10 μm) is necessary for Ca^2+^binding to all EF-hand domains (Park *et al.*, 2008) and subsequent fast hippocalcin translocation. Whatever the reason, the translocation following a single synaptic stimulation was negligible, effectively filtering subthreshold synaptic activity. Threshold EPSPs with 1-2 APs riding on the top of these EPSPs (Fig. 7A) did not produce any visible hippocalcin translocation to spines. Thus, the biophysical properties of hippocalcin are ideally suited to skip a single quantum release at the spine level. At the same time, bursting activity, which should result in a general increase of [Ca^2+^]_i_ in both dendritic shaft and spines, produced strong hippocalcin translocation to spines. A higher affinity for hippocalcin binding to the plasma membrane and larger [Ca^2+^]_i_ increase in a spine head compared with a dendritic shaft (Sabatini *et al.*, 2002) might be suggested as potential biophysical mechanisms for the observed spine specificity of hippocalcin insertion in the spine plasma membrane during a strong synaptic input (Fig. 7B). Hippocalcin depletion from the spine head cytosol after its insertion into the spine plasma membrane underlies its diffusion from a parent dendrite via a spine neck (equilibration time constant of about 100 ms) and its additional insertion in the spine membrane observed as an increase in spine hippocalcin concentration (Fig. 7B). Finally, iontophoretic glutamate application that activated the whole set of both synaptic and extrasynaptic glutamate receptors should induce a large and prolonged [Ca^2+^]_i_ increase in dendrites and strong hippocalcin translocation to the dendritic plasma membrane. This results in depletion of the cytosolic hippocalcin fraction in a dendritic shaft, preventing hippocalcin translocation to the spines (Fig. 7D). Such competition for a limited amount of hippocalcin among its targets can potentially produce a variety of complex effects, including dependence of particular hippocalcin-related activity on the history of hippocalcin translocation to disparate targets.

### Possible mechanisms of hippocalcin signalling in neurons

Based on the described biophysical properties, hippocalcin may contribute to intracellular signalling in several ways. Its site-specific Ca^2+^-dependent translocation to the plasma membrane may result in regulation of membrane-bound targets spatially segregated in the translocation sites. The higher hippocalcin affinity for membranes of certain composition (O'Callaghan *et al.*, 2005) as well as some protein–protein interactions with possible membrane targets (Haynes *et al.*, 2006; Tzingounis *et al.*, 2007; Tzingounis & Nicoll, 2008) may underlie this type of signalling. A direct interaction of hippocalcin with membranous targets under this scenario may be suggested when hippocalcin gates [Ca^2+^]_i_-dependent potassium channels responsible for slow (Tzingounis *et al.*, 2007) and medium (Tzingounis & Nicoll, 2008) afterhyperpolarization to regulate neuronal membrane potential and bursting activity.

At the same time, hippocalcin might signal as a ‘shuttle’ delivering specific molecules to their sites of action. At low [Ca^2+^]_i_, hippocalcin is probably folded with its N-terminal myristoyl group hidden in a hydrophobic pocket as described for the NCS protein recoverin (Ames *et al.*, 1997). The binding of Ca^2+^would result in a conformational switch and protrusion of the N-terminal region out of the pocket. The N-terminus or the exposed hydrophobic region of hippocalcin may interact with cytosolic target proteins in a Ca^2+^-dependent manner (Palmer *et al.*, 2005). Hippocalcin translocation to certain plasma membrane sites would bring the interacting proteins to their sites of action. Ca^2+^unbinding from hippocalcin would result in hippocalcin dissociation from the interacting protein and its translocation back to the cytosol. If [Ca^2+^]_i_ is still high the next round of shuttling would start. Fast and transient hippocalcin translocation to dendritic spines is a biophysical basis for this scenario. It has also been shown that hippocalcin can directly interact in a Ca^2+^-dependent manner with several neuronal cytosolic proteins (Haynes *et al.*, 2006), including those contributing to NMDAR-dependent AMPAR LTD (Palmer *et al.*, 2005). Although there are Ca^2+^-independent mechanisms of AMPAR LTD (Dickinson *et al.*, 2009), hippocalcin is specifically required for this Ca^2+^-dependent form of plasticity. Its N-terminal interaction with a cytosolic protein, the β2-adaptin subunit of the AP2 adaptor complex, is necessary for LTD expression (Palmer *et al.*, 2005). The hippocalcin-AP2 complex interacts with AMPARs, leading to AMPAR endocytosis via clathrin-coated vesicles. Moreover, hippocalcin is absent from these vesicles, indicating that the interaction between hippocalcin and the AP2-AMPAR complex is transient and may only occur at the plasma membrane, supporting the idea of the ‘shuttling’ function of hippocalcin at least in this signalling pathway. Fast off-rates of Ca^2+^binding to EF-hand-type proteins are also in line with this suggestion (Bayley *et al.*, 1984).

Finally, after activation of the Ca^2+^-myristoyl switch, hippocalcin has a high affinity for a phospholipid of the plasma membrane, PIP_2_ (O'Callaghan *et al.*, 2005), which is a messenger and precursor of other messenger molecules. Bearing in mind the high hippocalcin concentration in the hippocampus (about 30 μm; Furuta *et al.*, 1999) it could effectively buffer PIP_2_ in the plasma membrane, locally regulating PIP_2_-dependent intracellular signalling.

In conclusion, our data reveal that synaptic AMPAR and NMDAR activation, AP firing and extrasynaptic glutamate receptor activation produce different spatiotemporal patterns of hippocalcin translocation. We suggest that hippocalcin may differentially decode activation of glutamate receptors converting it to site- and time-specific modification of its targets. Hippocalcin may also process information in parallel in many sites within a neuron or produce local site-specific signalling at the level of single synapses.

## Abbreviations

AMPAR: AMPA receptor
AP: action potential
APV: d-2-amino-5-phosphonopentanoic acid
bpAPs: backpropagating action potentials
CFP: cyan fluorescent protein
CNQX: 6-cyano-7-nitroquinoxaline-2,3-dione
CPA: cyclopiazonic acid
EPSP: excitatory postsynaptic potential
ER: endoplasmic reticulum
gabazine: 6-imino-3-(4-methoxyphenyl)-1(6H)-pyridazinebutanoic acid
hippocalcin-YFP: yellow fluorescent protein-tagged hippocalcin
LTD: long-term depression
MCPG: α-methyl-4-carboxyphenylglycine
NCS: neuronal calcium sensor
NMDAR: NMDA receptor
ROI: region of interest
sAHP: slow afterhyperpolarization
VOCC: voltage-operated calcium channel
IP_3_: inositol 1,4,5-triphosphate
PIP_2_: phosphatidylinositol (4,5)-bisphosphate

## Supporting Information

Movie S1. Activation of synaptic NMDARs induces hippocalcin-YFP translocation to dendritic spines.

Fig. S1. VOCC activation did not result in hippocalcin-YFP translocation to dendritic spines.

Please note: As a service to our authors and readers, this journal provides supporting information supplied by the authors. Such materials are peer-reviewed and may be re-organized for online delivery, but are not copy-edited or typeset by Wiley-Blackwell. Technical support issues arising from supporting information (other than missing files) should be addressed to the authors.
