# Supplementary figures and images for: Decoding glutamate receptor activation by the Ca2+ sensor protein hippocalcin in rat hippocampal neurons
Source: Eur J Neurosci. 2010 Aug;32(3):347–58. doi: 10.1111/j.1460-9568.2010.07303.x (PMC3069492; doi:10.1111/j.1460-9568.2010.07303.x)

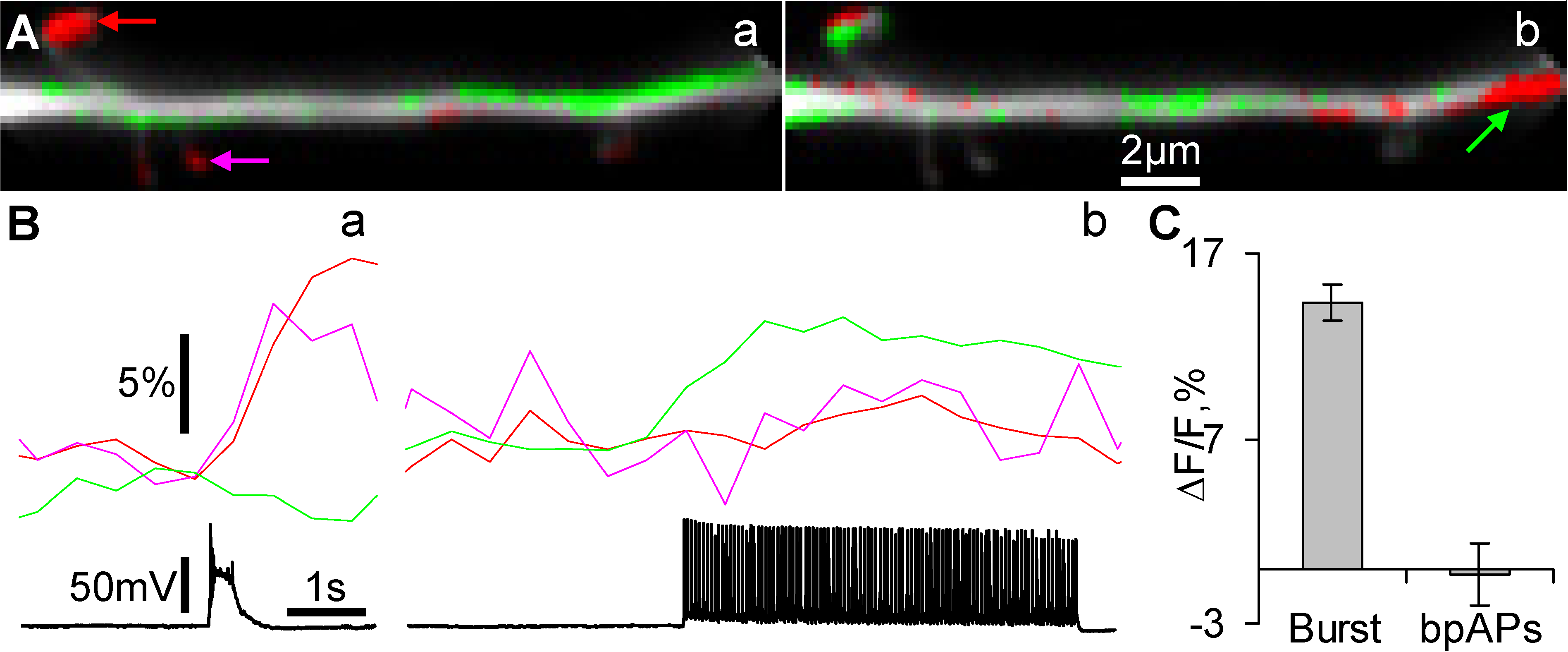

Supplement: Supplementary file 2 [file ejn0032-0347-SD2.tif]
